# Conserved gene regulation during acute inflammation between zebrafish and mammals

**DOI:** 10.1038/srep41905

**Published:** 2017-02-03

**Authors:** G. Forn-Cuní, M. Varela, P. Pereiro, B. Novoa, A. Figueras

**Affiliations:** 1 Inmunología y Genómica, Institute of Marine Research (IIM), Spanish National Research Council (CSIC), c/Eduardo Cabello, 6, 36208, Vigo, Spain

## Abstract

Zebrafish (*Danio rerio*), largely used as a model for studying developmental processes, has also emerged as a valuable system for modelling human inflammatory diseases. However, in a context where even mice have been questioned as a valid model for these analysis, a systematic study evaluating the reproducibility of human and mammalian inflammatory diseases in zebrafish is still lacking. In this report, we characterize the transcriptomic regulation to lipopolysaccharide in adult zebrafish kidney, liver, and muscle tissues using microarrays and demonstrate how the zebrafish genomic responses can effectively reproduce the mammalian inflammatory process induced by acute endotoxin stress. We provide evidence that immune signaling pathways and single gene expression is well conserved throughout evolution and that the zebrafish and mammal acute genomic responses after lipopolysaccharide stimulation are highly correlated despite the differential susceptibility between species to that compound. Therefore, we formally confirm that zebrafish inflammatory models are suited to study the basic mechanisms of inflammation in human inflammatory diseases, with great translational impact potential.

Understanding the molecular pathways governing inflammatory signaling is essential for advancing our knowledge of inflammatory-related human diseases. To this end, cultured immune cells and murine models are often used to control and study the mammalian acute inflammatory response^1^. These animal models have provided fundamental clues to understanding the mechanisms underlying human immunology and diseases, but recently, even murine models, the standard for inflammatory research, have been questioned as valid systems for the study human inflammatory diseases^1^,^2^. As a consequence, we sought to investigate if the zebrafish, an emerging animal model which has been proposed as appropriate to study vertebrate immunology^3^,^4^,^5^ and human diseases^6^,^7^,^8^,^9^,^10^, could reliably mimic human inflammatory diseases.

The advantages of using zebrafish for inflammatory research are numerous: small size, relatively rapid life cycle, external embryo maturation and transparency, ease of breeding and genetic manipulation, and a highly developed vertebrate immune system. However, there are noticeable differences between the mammalian and zebrafish immune responses, product of the evolutionary divergence, pathogen adaptation, and asymmetric evolution after the Fish Specific Genome Duplication^11^,^12^. One of these differential aspects is in the susceptibility to bacterial lipopolysaccharide (LPS – also known as endotoxin), which is routinely used to induce strong acute inflammation in mammalian studies^13^.

In mammals, LPS is specifically recognized by the TLR4-complex, a sensing receptor that is not evolutionarily conserved in fish^14^. While some fish species appear to have multiple copies of the *TLR4* gene, some of them soluble^15^, several sequenced fish genomes, such as *Tetraodon nigroviridis, Oryzias latipes*, and *Scophthalmus maximus*, lack TLR4 and its adapter proteins CD14 and MD2^16^,^17^,^18^, suggesting an alternative LPS-recognition pathway in fish. As in other fish, our knowledge of the recognition and signaling of LPS in zebrafish is far from complete. Although two TLR4 paralogs have been described in this species (*tlr4ba* and *tlr4bb*), they do not recognize LPS, likely due to differences in the extracellular structure^19^,^20^. Moreover, the reports for LPS signaling in zebrafish are unclear: it is described to signal both through *myd88*-independent^19^ and *myd88*-dependent^21^ pathways, but its detoxification and expression of proinflammatory markers appear to be *myd88*-dependent^21^,^22^. Regardless, zebrafish elicits a powerful inflammatory response to high LPS doses that can cause sepsis and death^22^,^23^,^24^,^25^ and reproduces certain mammalian characteristics, such as endotoxin tolerance^23^. Strikingly, a comprehensive description of the pathways and modulators activated during the LPS-induced acute inflammatory response and its correlation to mammalian inflammatory models to validate this zebrafish model is lacking.

In this work, we have analysed the transcriptomic modulations of different zebrafish tissues to LPS. The objective was to characterize the zebrafish genomic responses after an acute endotoxin stress event and determine whether they can accurately reproduce the mammalian acute inflammatory process despite the alternative, and currently uncertain, LPS-recognition mechanism. In this process, we sought to evaluate the zebrafish as a well-correlated and reliable model for in-depth acute inflammatory research.

## Results

### General transcriptomic modulation of the zebrafish inflammatory response

To analyse the general transcriptomic modulation during the zebrafish acute inflammatory response, kidney, liver and muscle were sampled from 9 month old adult zebrafish 3 hours after LPS intraperitoneal injection, and gene expression was investigated using commercial microarrays. As a first approach to analyse the overall response to the stimulus, the microarray data obtained from the three tissues were explored as a whole and compared to characterized gene sets involved in immune responses using Gene Set Enrichment Analysis (GSEA) and the MSigDB (Molecular Signatures Database) C7 (*immunological signatures*) database (Supplementary Table 1A), which confirmed the resemblance between the gene modulation in zebrafish and the ones elicited in mammalian immune responses. In order to determine the main common genes between our data and the significantly similar gene sets, we used a leading-edge analysis^26^. Homologs to mammalian inflammatory responsive genes, such as *il1b, atf3, myd88, irak3* or *junba*, were found to be a biologically active subset of genes in these phenotypes, hinting at the involvement of typical mammalian inflammatory signaling pathways on the zebrafish response to LPS. In fact, mapping the gene modulation to different pathways using the curated MSigDB C2 (*canonical pathways*) database in GSEA resulted in significant enrichment for immune-related pathways, such as antigen detection and processing (e.g., *KEGG Toll-Like Receptor Signaling*), immune signaling (e.g., *Reactome NFKB and MAP Kinases Activation Mediated by TLR4 Signaling Repertoire*) and apoptosis (e.g., *Biocarta Caspase Pathway*).

We complemented the gene enrichment analysis studying single genes with statistically differential expression in response to the inflammatory stimulus. Overall, 128 characterized genes were found to be significantly regulated 3 hours after the LPS injection across all studied tissues. The majority of the most significantly modulated genes were well-recognized inflammatory effectors, such as interleukins (*il1b, cxcl8a, il6*), chemokines (*cxcl11.1, cxcl18b, ccl34a.4*), and anti-inflammatory agents (*atf3, socs1b*) (Supplementary Table 2A).

### Tissue-specific transcriptomic modulation to LPS

To better understand the zebrafish inflammatory responsiveness to acute LPS stimulus, we analysed the specific transcriptomic modulation of individual tissues and how it contributed to the overall gene modulation. In concordance with the above results, comparison of the gene expression in response to LPS from each tissue to the MSigDB C7 (*immunological signatures*) to search for gene enrichment produced significant results with datasets from mammalian LPS stimulated samples (Supplementary Table 1B-D), though the level and significance of the enrichment were variable. In this regard, the kidney –the main hematopoietic tissue in fish– was the tissue with most number of significant results in the enrichment with immunological datasets, whereas liver and muscle tissue had lower immunological enrichment scores.

**Figure 1:**
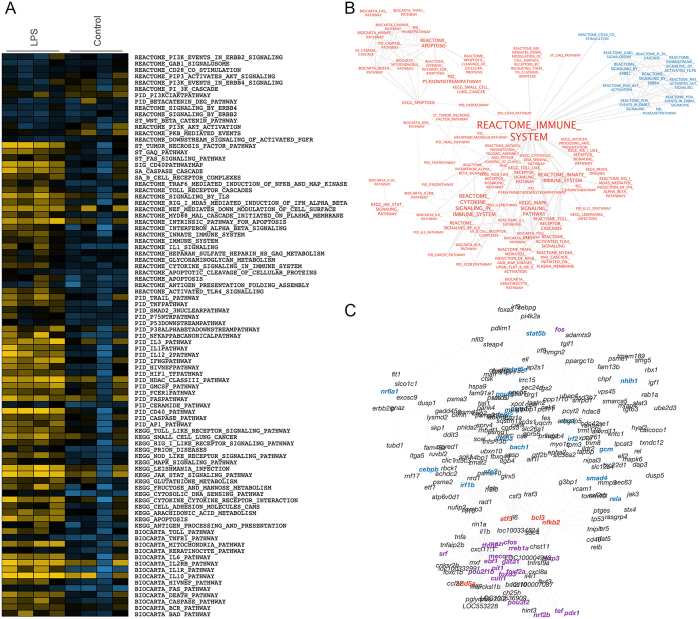
(**A**) Pathways enriched in the kidney transcriptomic response to LPS. ssGSEA was used to determine the degree to which the gene sets of the different pathways were coordinately upregulated (yellow) or downregulated (blue) in each microarray sample in response to LPS or PBS (Control). (**B**) Visualization of the pathways enriched in the kidney response to LPS. In red, the gene sets enriched in the LPS phenotype; in blue, the gene sets enriched in the control are shown. The label size is proportional to gene set size. The edge width is proportional to the number of common genes between the gene sets. (**C**) Network of transcription factors regulating the kidney gene expression in response to LPS. In red, the transcription factors differentially expressed after the LPS administration are shown. In purple, the transcription factors derived from the expression of differentially expressed genes are shown. In blue, the transcription factors based on the enrichment of their targets in GSEA are shown.

Pathway modulation analysis in the kidney correlated well with the general overview, meaning that there was significant enrichment of genes belonging to different inflammatory signaling routes and apoptosis in the LPS-stimulated kidney (Fig. 1A,B). Additionally, 45 genes, the majority of which being cytokines and growth factors (i.e., *ccl-c25y, ccl34.a, ccl35.2, ccl22, csf3, cxcl8a, cxcl11.1, il10, il1b, il6, tnfa*), were found to be statistically upregulated in the stimulated kidney, while only *cnksr2* was found down-regulated (Supplementary Table 2B). Up to 48 transcription factors were classified as putatively active and orchestrating gene expression during the zebrafish inflammatory response in the kidney based on the combined information of the significantly modulated genes and the enrichment of their targets (Fig. 1C, Supplementary Table 3).

In the liver, enrichment analysis of pathways and transcription factor targets produced no significant results. The absence of gene enrichment contrasted with the fact that the liver had 125 significant differentially expressed genes 3 hours after the LPS stimulation, which was the highest number in the three tissues. Most of the differentially expressed genes were categorized as immune signaling and apoptosis genes (Supplementary Table 2C). For example, similar to the kidney, a high number of cytokines (*ccl19a.1, ccl20a.3, ccl34.4, ccl35.2, ccl39.1, cxcl8a, cxcl11.1, cxcl18b, ifng1-2, il1b, il10, il34, tnfa*) and apoptosis-related genes (*card9, casp9, cflara, diabloa*) were modulated.

Concerning the muscle tissue alone, no differentially expressed genes were found to be statistically significant after the LPS injection. Nevertheless, GSEA determined that there was significant enrichment of the genes related to the *Signaling Gateway CD40 Pathway* (Normalized Enrichment Score = 1.88; p-value = 0.000; FDR = 0.206) and of the HOXA5 transcription factor targets in the stimulated muscle (Supplementary Table 3). Though none of the genes were statistically significant at the selected FDR cut-off, the upregulation of gene sets belonging to immune signaling pathways and the significant enrichment of datasets from LPS stimulated tissues in GSEA revealed that the inflammatory response in the muscle was present, albeit not as strong as in the other analysed tissues.

### Comparison between the zebrafish and the mammalian response to LPS

**Figure 2:**
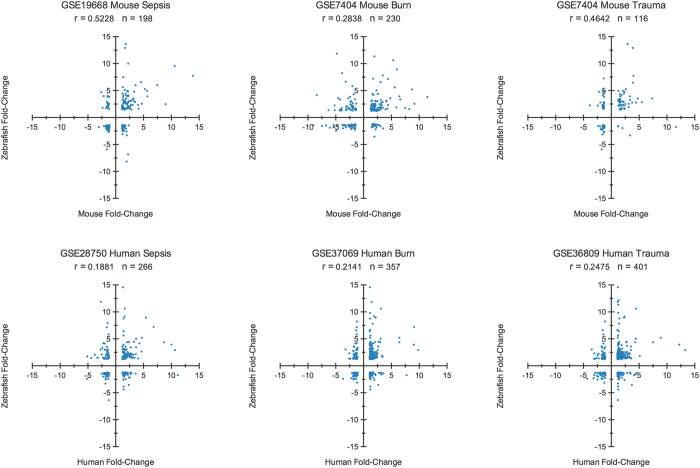
Scatter plot of the correlation between zebrafish LPS response mammal inflammatory diseases. The gene expression modulation of common significant genes in the zebrafish LPS response and mammal inflammatory diseases (Sepsis, Burn, Trauma) was evaluated. Although all of the correlations were significant (p < 0.01 in all), correlation values were variable between comparisons.

To investigate the similarities and differences between the zebrafish and mammalian response to LPS, we analysed the correlation between differentially expressed homolog genes in zebrafish and mice using the nonparametric Spearman’s correlation coefficient. Similarly to previous works, we compared the gene modulation in our zebrafish model to mammal inflammatory diseases, considering the correlation of genes significantly differentially expressed in human or mouse regardless of their significance in zebrafish^2^ (Supplementary Fig. 1) as well as only genes that were significantly differentially expressed in both species^1^ (Fig. 2). Although significant, all correlations were poor when considering zebrafish homologs regardless of their statistical significance in both species studied but greatly improved when considering only significant genes in both species. Due to the similarity in their stimulus, the zebrafish LPS response and the mouse sepsis model was the highest correlated comparison.

**Figure 3:**
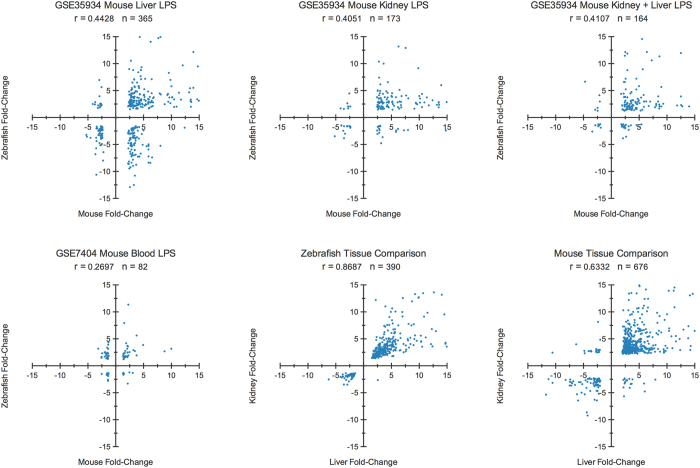
Scatter plot of the correlation between zebrafish and mouse genomic response to LPS. The gene expression modulation of common genes in the kidney, liver and both tissues shortly after LPS stimulation between zebrafish and mouse was highly correlated. However, correlation between these tissues and blood derived white-cells was poor. Gene expression correlation between the liver and kidney was also found in zebrafish and mouse. p < 0.01 in zebrafish–mouse blood correlation, p < 0.0001 in rest.

To highlight the importance of properly mimicking the conditions of inflammatory diseases in animal models, we also compared the gene modulation between similar mouse and zebrafish conditions, tissues and timing. Shortly after intraperitoneal LPS stimulation, the differentially expressed genes in the liver, kidney, and both tissues together between the zebrafish and mouse LPS response were found to be highly similar (Fig. 3). In fact, approximately 84% of the homolog genes in the kidney and 68% in the liver retained directionality between the two species. Interestingly, most of the homolog genes were commonly upregulated in both species and across tissues in the same species. In addition, tissue-specific modulations were maintained between the two species. For example, the cytokines *Ccl20 (ccl20a.3*) and *Ifng (ifng1-2*) were only significantly modulated in the liver, while *il4i1* was only modulated in the kidney of the mouse and zebrafish. Importantly, directly comparing gene modulation between our studied zebrafish tissues and mouse LPS-stimulated white blood cells resulted in poor gene correlation values.

**Figure 4:**
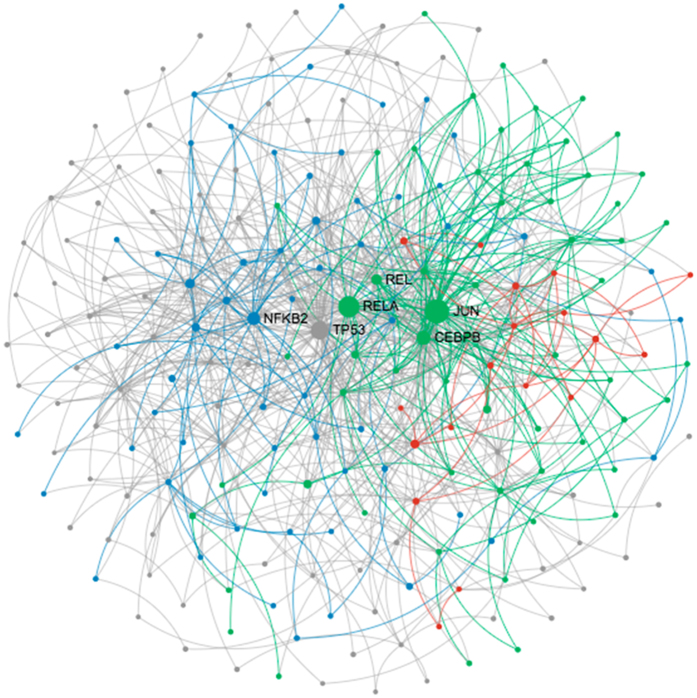
Network of evolutionary conserved genes in response to LPS. The transcription factors putatively conserved between the adult zebrafish and mouse in response to LPS stimulation were analyzed in NetworkAnalyst to produce a network of potential genes with evolutionarily conserved expression across species in which genes are shown as circles. NetworkAnalyst predicted three different significant modules in the network related to immune and stress signaling (blue), apoptosis and cellular cycle (green) and cytokine production and signaling (red).

Finally, we directly compared the overall modulation using datasets from LPS-stimulated mouse, human and zebrafish tissues. The gene sets from mouse and zebrafish were highly bidirectionally ranked in GSEA, confirming the resemblance between the zebrafish and mammalian response to LPS as seen in the correlation studies (Table 1). On a pathway level, 75% of the gene sets from pathways determined to be significant during zebrafish inflammation were commonly enriched in the mouse following LPS stimulation. Moreover, 23 potential transcription factors were found to be common between the two species and were imported into NetworkAnalyst to create a network of evolutionary conserved gene regulation in response to LPS. NetworkAnalyst significantly differentiated three modules in the network: the response to stress and intracellular immune signaling, apoptosis and cellular cycle, and cytokine regulation and production (Fig. 4).

## Discussion

Due to controversial reported differences in gene modulation during inflammatory stimulation, the use and applicability of murine models, and consequently of animal inflammatory models in general, has been questioned over the past few years^1^,^2^. At the same time, the zebrafish is gaining popularity as a model for studying human diseases and vertebrate immunology. However, if the use of murine models for studying human inflammation processes is questioned, how relevant can a more evolutionarily distant species such as zebrafish be for modelling human diseases? More importantly, the whole zebrafish transcriptomic response during acute inflammation and its correlation to the mammalian response is still unknown.

In this study, we demonstrate how gene modulation in zebrafish after administration of LPS, an inflammatory bacterial compound without evolutionary conserved detection mechanism and susceptibility^13^,^14^ is in concordance to that of mammals. As a consequence, we concur with the previous observation that despite the physiological differences between species and the altered susceptibility and responsiveness to specific pathogens or inflammatory inducers, inflammatory signaling is largely conserved across evolution^13^, and zebrafish inflammatory models can successfully represent human inflammatory responses.

We characterized the zebrafish inflammatory response both by analysing genes with statistically significant fold-changes and by systematically using a gene-ranking algorithm, GSEA, which allows for the comparison of microarray data and gene lists from different experiments and across different species to determine the similarities^26^. Similar to mammals, the zebrafish transcriptomic response to the inflammatory stimulus affected primarily genes associated with the detection, processing and signaling of external antigens, immune defence pathways and apoptosis.

The basal condition of different tissue types greatly affects specific gene regulation, as the same gene in different cell types may produce unexpected or even opposite functions^27^. To account for these variables, we compared the differential transcriptomic response to LPS between three distinct representative zebrafish tissues: the kidney, the main hematopoietic tissue in fish with well-recognized immune functions in teleosts; the liver, an organ serving mainly metabolic functions but with an infiltrated immune cell population; and the muscle, which has limited immune functions. While every tissue correlated well with the overall modulation of immunologically enriched gene sets, the strength and significance of the correlation for the expression of specific genes varied between the tissues. In concordance with their expected functions, we found that the kidney tissue was the most enriched in immune pathways and interactors, and the liver had most differentially expressed genes but less immune enrichment. We could not identify any differentially expressed gene in the muscle at the restrictive significance levels used, but GSEA determined that there was a tendency to increase gene expression of the immune-related CD40 signaling pathway.

Finally, we directly compared the overall transcriptomic modulation following LPS treatment between zebrafish and mammals. At this point, it is necessary to emphasize the current problems with establishing homologous relationships between distant species, in this case, between the zebrafish and mammal genomes. Since the first early drafts of the zebrafish genome became available, significant advances to the genome quality have been made and new genomes of species positioned evolutionarily between zebrafish and humans have been sequenced. In addition, computational methods are being developed to aid with comparative genomics. However, homology prediction for the majority of the zebrafish genome is still based on sequence similarity and not functional characterization, which may be prone to error^28^,^29^. Two inflammation-related examples are the *tlr4ba* and *tlr4bb*, and the *c3b.1-2* zebrafish genes, which were predicted to be orthologs of human *TLR4* and *C3* until in depth studies proposed that these genes were ohnologs^20^ and paralogs^30^, respectively.

Moreover, as discussed below, LPS susceptibility is not evolutionary conserved between the studied species^13^, mainly due to the absence in fish of a functional highly-specific TLR4-mediated LPS detection system as in mammals^14^. In consequence, fish are generally regarded as less sensible to LPS^9^. However, the concentration of intraperitoneally injected LPS used to achieve the endotoxemia state in zebrafish (10 mg/kg of *E. coli* 0111:B4 in this and previous studies), is the same as in other murine models^31^,^32^,^33^. As the required LPS doses to promote sepsis depends on the LPS bacterial origin species, serotype, the administration method, and the specific animal conditions and strains^34^,^35^,^36^, caution must to be taken when comparing LPS doses between animal models.

Strikingly, despite the aforementioned discrepancies, we report how specific gene regulation is well conserved across evolution in inflammatory diseases. The Spearman’s correlation coefficient test of differentially expressed homologs resulted in significant correlation levels between zebrafish LPS response and mammal inflammatory diseases, especially in the response to a similar insult: sepsis and purified LPS. Although it is true that, as Seok *et al*. reported^2^, correlations are poor when considering the modulation of genes significantly expressed in only one animal regardless of their significance in the other model, we agree with Takao *et al*. in that to detect the true responses shared in the animal model, only genes significantly differentially expressed in both species must be considered^1^.

Moreover, our analysis evidences that, to successfully obtain high correlation between an animal model and the condition it represents, the environment has to be carefully mimicked. In consequence, we compared the gene modulation of similar zebrafish and mouse shortly after LPS stimulation, obtaining high correlation values of 0.40 to 0.44. Considering that reported correlation values between human and mouse models for inflammatory diseases range between 0.43 and 0.68^1^, we can affirm that the modulation of gene homologs between the zebrafish and mammals is highly conserved. It is noteworthy that not only were the majority of gene homologs across all of the comparisons upregulated in both species in response to the inflammatory stimulus (50–75%) but the same behaviour was also accentuated between both mouse and zebrafish tissues (83–84%).

Finally, independently of the comparison of specific homolog genes, we could positively correlate the overall gene modulation of the zebrafish, mouse and human datasets after LPS stimulation. In fact, we found a high degree of conservation between the zebrafish and mammal transcription factors and pathway signaling in response to LPS. These results confirm previous observations that, although the specific components assumed to interact with the pathogens are more divergent, the intracellular signaling components in vertebrates are highly conserved^37^.

Zebrafish models possess many of the advantages of invertebrate models while also containing a highly developed immune system, which allows for easy visualization of *in vivo* inflammatory processes in a whole-animal context and relatively easy high-throughput analyses. Consequently, it is not difficult to understand why the zebrafish repeatedly has been proposed as a promising model for the study of basic and human immunology and many human diseases are being modelled in this animal^8^. However, animal models can only reproduce certain aspects of human disorders, and there are many challenges on the translational impact of inflammatory animal models that will only be solved by correctly mimicking the conditions of each disease^38^. In summary, we validated the use of the LPS-stimulated zebrafish model to study acute inflammatory signaling in mammal diseases, demonstrating how acute inflammatory signaling and its transcriptional mechanisms are conserved between zebrafish and mammals despite different pathogen susceptibility and recognition. This model includes a diverse investigatory toolbox for visualization and screening approaches to further understand innate immunity and inflammatory diseases that may be exploited while taking careful consideration of the environment and application for translational purposes.

## Methods

### Animals

Zebrafish were obtained from our experimental facilities where zebrafish are cultured following established protocols^39^,^40^. All experimental procedures followed Spanish Law for Animal Experimentation (Royal Executive Order, 53/2013), in accordance with European Union directive 2010/63/UE. Fish care and challenge experiments were reviewed and approved by the CSIC National Committee on Bioethics (approval number: 01_09032012). Adult (9 month) *wild-type* fish were intraperitoneally injected with 10 μg of LPS (Sigma L2630) or an equal volume of PBS. For each treatment and tissue (liver, kidney or muscle), 4 biological replicates (pools of 3 fish/replicate) were sampled 3 hours post-injection and stored at −80 °C until use for the microarray analysis.

### RNA isolation and cDNA transcription

RNA was extracted with TRIzol reagent (Life Technologies, Madrid, Spain) following the TRIzol manufacturer’s specifications in combination with the RNeasy Mini Kit (Qiagen, Madrid); the extracted RNA was preserved at −80 °C until use. After DNase I treatment, 1 μg of total RNA was used to obtain cDNA using the SuperScript III First-Strand Synthesis SuperMix for qRT-PCR (Life Technologies, Madrid, Spain).

### Microarray analyses

The 4 × 44 K Zebrafish Gene Expression Microarray (V3, AMADID 026437) containing 43,803 probes representing 23,207 genes was used (Agilent Technologies; Madrid, Spain). RNA quality was assessed with the Agilent 2100 Bioanalyzer and stored frozen at −80 °C until all of the RNA could be hybridized and processed simultaneously. The labelling of 2 μg of RNA (~50 μg/ml) and hybridization were carried out using the Universidad Autónoma de Barcelona microarray platform, complying with the Minimum Information about a Microarray Experiment (MIAME) standards. The signal was captured, processed, and segmented using an Agilent G2565B scanner (Agilent Technologies, Madrid, Spain) with Agilent Feature Extraction Software (v9.5) protocol GE1-v5_95 using an extended dynamic range and preprocessing by Agilent Feature Extraction v9.5.5.1.

The results for the fluorescence intensity data and quality annotations were imported into GeneSpring GX version 12.6 (Agilent Technologies). Normalized microarray data from each tissue is publicly available at GEO under Series Accession ID GSE73223. All of the control features (including the positive and negative controls and the landing lights) were excluded from subsequent analyses. Normalization was then carried out by a percentile shift at the 75^th^ percentile. Entities with an expression between the 20^th^ and 95^th^ percentiles in the raw data were retained and used in subsequent analyses. To assess genes for differential expression, the normalized log intensity ratios were analyzed with a Moderated T-test with Benjamini-Hochberg FDR multiple testing correction, and significance was established at a corrected p < 0.05. Microarray expression values were validated with qPCR expression analysis of 6 different genes.

The microarray results were compared to Molecular Signatures Database (MSigDB) sets and other previously published inflammatory models using Gene Set Enrichment Analysis (GSEA)^26^. Although there were less than 8 samples for each treatment, we kept the permutation_type parameter at a phenotype level in order to generate a more robust and restrictive analysis. ssGSEA was used to determine the degree at which the gene sets were co-ordinately upregulated or downregulated in each sample. Visualization of the transcription factor networks was performed using Cytoscape v3.0.2. The pathway networks from the GSEA results were visualized with the Cytoscape plugin Enrichment Map^41^.

For correlation analysis, significance was considered without multitest correction as in previous studies^1^,^2^. Raw microarray data of mammalian inflammatory diseases referenced in the compared studies^1^,^2^ were obtained from the GEO online repository. Similarly, mouse tissue microarray data in response to LPS were obtained from published studies^42^. To maintain consistency, all the data were reanalysed using the same parameters used throughout this study. In all cases, the most significantly correlated comparison of conditions (tissue, time post stimulation, etc.) was chosen. Homolog gene data was obtained from bioDBnet^43^ using microarray annotated Entrez Gene IDs. Curated LPS-stimulated datasets were retrieved from published studies: mouse liver and kidney (GSE35934)^42^ and bone marrow derived macrophages (GSE14769)^44^; human-derived monocytes (GSE9988)^45^ and dendritic cells (GSE14000)^46^. The datasets can be found in Supplementary Table 4. The interactions between the evolutionary conserved transcription factors were investigated using NetworkAnalyst^47^.

## Additional Information

**How to cite this article**: Forn-Cuní, G. *et al*. Conserved gene regulation during acute inflammation between zebrafish and mammals. *Sci. Rep.* **7**, 41905; doi: 10.1038/srep41905 (2017).

**Publisher's note:** Springer Nature remains neutral with regard to jurisdictional claims in published maps and institutional affiliations.

## Supplementary Material

Supplementary Information

Supplementary Table 1

Supplementary Table 4

## Figures and Tables

**Table 1:** Results from the bidirectional GSEA comparison between LPS-stimulated mouse and zebrafish tissues and other LPS datasets.

Investigated dataset	Species	Mouse Dataset			Zebrafish Dataset		
		NES	p-value	FDR	NES	p-value	FDR
Upregulated in liver stimulated with LPS	Zebrafish	2.34	0	0	1.92	0	0.002
GSE14000 - Translated RNA downregulated in unstimulated DCs vs 4 h LPS	Human	2.26	0	0	2.01	0	0.001
GSE14000 - Downregulated in unstimulated DCs vs 4 h LPS	Human	2.21	0	0	1.99	0	0.001
GSE9988 – Upregulated in monocytes after LPS vs vehicle.	Human	2.16	0	0	2.32	0	0
Upregulated in kidney, liver and muscle LPS vs control	Zebrafish	2.12	0	0	1.99	0	0.001
Upregulated in kidney stimulated with LPS	Zebrafish	2.04	0	0.001	1.88	0	0.003
GSE14769 – Downregulated in unstimulated Bone Marrow Derived Macrophages vs 120 min LPS	Mouse	1.96	0	0.002	2.29	0	0
GSE35934 - Upregulated in liver stimulated with LPS	Mouse	1.92	0	0.003	2.35	0	0
GSE35934- Upregulated in kidney stimulated with LPS	Mouse	1.92	0.002	0.003	2.22	0	0

NES = Normalized Enrichment Score, FDR = Benjamini-Hochberg False Discovery Rate.
