# Supplementary material for: Conserved gene regulation during acute inflammation between zebrafish and mammals
Source: Sci Rep. 2017 Feb 3;7:41905. doi: 10.1038/srep41905 (PMC5291205; doi:10.1038/srep41905)
Supplement: Supplementary Information [file srep41905-s1.pdf]

Conserved gene regulation during acute inflammation between zebrafish and mammals

Forn-Cuní G., Varela M., Pereiro P., Novoa B., Figueras A.

Inmunología y Genómica, Institute of Marine Research (IIM), Spanish National Research Council (CSIC), c/Eduardo Cabello, 6, 36208, Vigo, Spain

Supplementary Information

Supplementary Figure 1

Scatter plot of the correlation between zebrafish LPS response mammal inflammatory diseases using all homolog genes. The gene expression modulation of all genes in the zebrafish LPS response and mammal inflammatory diseases (Sepsis, Burn, Trauma) despite of their statistical significance was evaluated. As expected, the correlation values were poor.

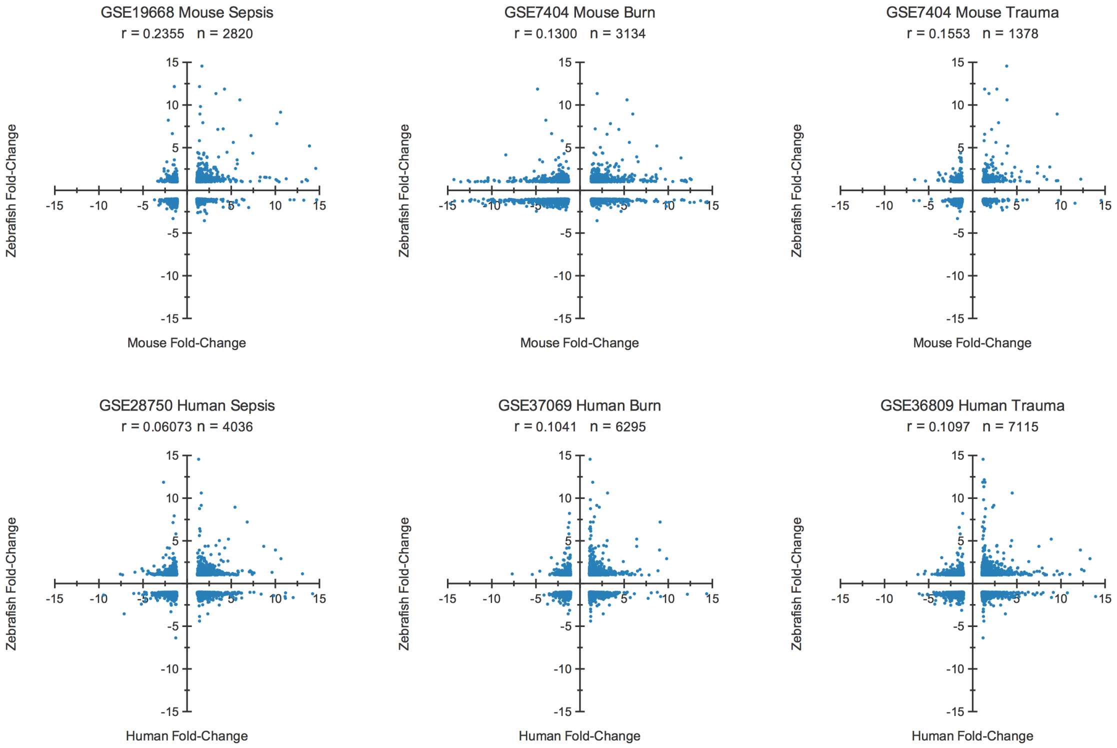

Supplementary Table 2

Gene list of the top 25 most-modulated genes 3 hours after intraperitoneal LPS injection across all tissues (A), in the kidney (B) and in the liver (C).

A. Top 25 most modulated genes in the general comparison between LPS-stimulated and control adult zebrafish.

Gene Symbol	Gene Name	Fold-Change	FDR
slc12a9	Danio rerio novel protein to vertebrate solute carrier family 12 (potassium/chloride transporters), member 9	52.33	3.40E-07
loc100007087	basic leucine zipper transcriptional factor ATF-like	49.86	3.40E-07
il1b	interleukin 1, beta	42.46	3.40E-07
loc100536909	poliovirus receptor-related protein 3-like	32.55	1.56E-06
mxf	myxovirus (influenza virus) resistance F	31.75	3.50E-04
cxcl11.1	chemokine (C-X-C motif) ligand 11, duplicate 1	29.78	3.07E-05
cxcl8a	chemokine (C-X-C motif) ligand 8a	17.55	1.07E-06
cxcl18b	chemokine (C-X-C motif) ligand 18b	16.67	8.86E-06
irg1	immunoresponsive 1 homolog (mouse)	16.14	1.03E-04
atf3	activating transcription factor 3	14.56	3.09E-04
ccl35.2	chemokine (C-C motif) ligand 35, duplicate 2	14.17	8.65E-04
loc553228	gamma-glutamyl hydrolase	14.03	6.21E-05
loc100000034	chemokine CCL-C25y-like	13.20	4.72E-04
si:ch211-284o19.8	protein lifeguard 1	12.36	0.040898103
si:dkey-40g16.6	L-amino-acid oxidase	12.15	5.49E-04
socs1b	suppressor of cytokine signaling 1b	11.97	0.001584599
tnfaip2b	tumor necrosis factor, alpha-induced protein 2b	11.97	3.21E-06
tnfa	tumor necrosis factor a (TNF superfamily, member 2)	11.87	8.64E-05
irf1b	interferon regulatory factor 1b	11.35	0.00315177
bcl3	B-cell CLL/lymphoma 3	10.60	0.003768386
lacc1	Laccase (multicopper oxidoreductase) domain containing 1	10.17	6.25E-07
ccl34a.4	chemokine (C-C motif) ligand 34a, duplicate 4	9.94	0.001755606
ch25h	cholesterol 25-hydroxylase	9.83	8.08E-05
il6	interleukin 6	9.64	0.001254349
loc100004948	TNFAIP3-interacting protein 1-like	9.56	0.002175428

B. Top 25 most modulated genes in the comparison between LPS-stimulated and control adult zebrafish kidney.

Gene Symbol	Gene Name	Fold-Change	FDR
il1b	interleukin 1, beta	142.92	3.42E-03
loc100007087	basic leucine zipper transcriptional factor ATF-like	119.91	5.60E-03
cxcl11.1	chemokine (C-X-C motif) ligand 11, duplicate 1	76.39	7.39E-03
loc100332991	Danio rerio novel protein to vertebrate solute carrier family 12 (potassium/chloride transporters), member 9	69.17	3.53E-02
loc100536909	poliovirus receptor-related protein 3-like	54.40	1.71E-02
loc553228	gamma-glutamyl hydrolase	50.69	2.77E-03
mxf	myxovirus (influenza virus) resistance F	43.71	2.28E-02
il6	interleukin 6	40.84	4.80E-02
il10	interleukin 10	36.09	8.62E-03
csf3	colony stimulating factor 3 (granulocyte)	34.07	3.53E-02
zgc:158404	similar to PLEKHS1	33.79	2.64E-02
bcl3	B-cell CLL/lymphoma 3	31.43	1.44E-02
cxcl8a	chemokine (C-X-C motif) ligand 8a	29.24	1.71E-02
tnfa	tumor necrosis factor a (TNF superfamily, member 2)	22.47	0.033771582
lacc1	Laccase (multicopper oxidoreductase) domain containing 1	21.91	7.39E-03
cxcl18b	chemokine (C-X-C motif) ligand 18b	21.32	0.00739431
pglyrp5	peptidoglycan recognition protein 5	19.41	7.39E-03
il4i1	interleukin 4 induced 1	19.39	1.69E-02
irf1b	interferon regulatory factor 1b	19.07	0.032731503
hint3	Histidine triad nucleotide-binding protein 3	18.86	0.032508053
ccl35.2	chemokine (C-C motif) ligand 35, duplicate 2	15.98	2.64E-02
chst11	carbohydrate (chondroitin 4) sulfotransferase 11	15.91	0.017057208
ch25h	cholesterol 25-hydroxylase	15.85	1.71E-02
snap23.2	synaptosomal-associated protein 23.2	15.77	0.031728234
cnksr2b	connector enhancer of kinase suppressor of Ras 2b	-15.54	0.032508053

C. Top 25 most modulated genes in the comparison between LPS-stimulated and control adult zebrafish liver.

Gene Symbol	Gene Name	Fold-Change	FDR
LOC100007087	basic leucine zipper transcriptional factor ATF-like	76.49	2.0E-04
LOC100536909	poliovirus receptor-related protein 3-like	69.80	9.3E-03
slc12a9	vertebrate solute carrier family 12 (potassium/chloride transporters), member 9	65.87	2.0E-04
il1b	interleukin 1, beta	46.24	2.5E-03
cxcl18b	chemokine (C-X-C motif) ligand 18b	44.76	3.5E-03
si:ch211-222m15.19	si:ch211-222m15.19	43.13	4.2E-03
atf3	activating transcription factor 3	43.10	1.7E-02
ccl20a.3	chemokine (C-C motif) ligand 20a, duplicate 3	42.49	1.2E-02
ccl35.2	chemokine (C-C motif) ligand 35, duplicate 2	35.72	2.5E-03
LOC100000034	chemokine CCL-C25y-like	32.75	2.5E-03
cxcl8a	chemokine (C-X-C motif) ligand 8a	31.96	7.7E-03
cxcl11.1	chemokine (C-X-C motif) ligand 11, duplicate 1	29.47	4.3E-03
noxo1a	NADPH oxidase organizer 1a	27.64	2.8E-02
irg1	immunoresponsive 1 homolog (mouse)	27.44	4.1E-04
snap23.2	synaptosomal-associated protein 23.2	26.71	3.4E-02
si:ch211-284o19.8	lifeguard 1	24.01	3.9E-02
ccl34a.4	chemokine (C-C motif) ligand 34a, duplicate 4	23.27	2.1E-02
diabloa	diablo, IAP-binding mitochondrial protein a	-23.01	8.3E-03
plekhf1	pleckstrin homology domain containing, family F (with FYVE domain) member 1	22.74	3.2E-02
mpeg1.2	macrophage expressed 1, tandem duplicate 2	22.46	1.0E-02
LOC567472	uncharacterized LOC567472	21.56	5.9E-03
tnip1l	TNFAIP3-interacting protein 1-like	19.54	8.3E-03
tlr5b	toll-like receptor 5b	17.17	2.9E-02
ch25h	cholesterol 25-hydroxylase	17.16	1.8E-02
irak3	interleukin-1 receptor-associated kinase 3	16.08	2.0E-02

Supplementary Table 3
Enrichment results for general, kidney, and muscle phenotypes using the *C3 TFT* (*Transcription Factor Targets*) MySigDB using GSEA.

Dataset (Transcription Factor)		All			Kidney			Muscle		
Name	Overlapping genes	NES	p-val	FDR	NES	p-val	FDR	NES	NOM p-val	FDR q-val
V$NFKB_C	65/263	2.075	0.000	0.005	1.939	0.000	0.015			
GGGNNTTTCC_V$NFKB_Q6_01	38/134	2.040	0.002	0.003	1.902	0.000	0.015			
V$NFKB_Q6_01	60/232	1.913	0.000	0.023	1.739	0.000	0.059			
V$NFKAPPAB65_01 (RELA)	61/237	1.888	0.002	0.024	1.872	0.000	0.015			
V$NFKB_Q6	76/254	1.839	0.000	0.040	1.685	0.000	0.111			
V$MEIS1BHOXA9_01 (MEIS1)	38/145	1.819	0.000	0.043	1.562	0.000	0.202			
V$GNCF_01 (NR6A1)	17/78	1.796	0.010	0.052	1.656	0.000	0.137			
V$NFKAPPAB_01 (NFKB RELA)	63/251	1.776	0.002	0.056	1.841	0.000	0.018			
V$CEBPB_01 (CEBPB)	70/262	1.705	0.002	0.126	1.742	0.000	0.065			
V$HOX13_01 (HOXA5)	15/46	1.705	0.010	0.114				1.710	0.000	0.182
V$CREL_01 (REL)	70/256	1.702	0.008	0.107						
V$POU6F1_01 (POU6F1)	57/240	1.653	0.002	0.161	1.562	0.000	0.199			
YWATTWNNRGCT_UNKNOWN	23/70	1.635	0.010	0.179	2.191	0.000	0.015			
TAAYNRNNTCC_UNKNOWN	40/172	1.634	0.010	0.167						
V$TATA_C (TAFIID)	75/283	1.594	0.008	0.230						
V$AP1_Q4_01 (JUN)	69/261	1.582	0.010	0.240	1.607	0.000	0.157			
V$IRF2_01 (IRF2)	39/125	1.573	0.016	0.245	1.751	0.000	0.056			
V$IRF1_01 (IRF1)	74/250				1.823	0.000	0.025			
V$NFE2_01 (NFE2)	80/272				1.720	0.000	0.086			
V$TEF1_Q6	55/226				1.646	0.000	0.128			
V$SMAD4_Q6 (SMAD4)	62/241				1.664	0.000	0.134			
V$GCM_Q2 (GCM1)	66/242				1.633	0.000	0.135			
V$STAT5A_01 (STAT5A)	75/251				1.646	0.000	0.136			
V$IRF1_Q6 (IRF1)	79/258				1.648	0.000	0.143			
V$STAT5B_01 (STAT5B)	72/247				1.600	0.000	0.163			
V$SPZ1_01 (SPZ1)	61/231				1.573	0.000	0.197			
V$BACH1_01 (BACH1)	71/263				1.576	0.000	0.201			
YKACATTT_UNKNOWN	78/276				1.566	0.029	0.202			
V$HEN1_02 (NHLH1)	60/198				1.529	0.000	0.246			
V$AP1_01 (JUN)	78/267				1.505	0.000	0.248			
